# Adherence to the Dutch healthy diet index and change in glycemic control and cardiometabolic markers in people with type 2 diabetes

**DOI:** 10.1007/s00394-022-02847-6

**Published:** 2022-03-14

**Authors:** Ehlana Catharina Maria Bartels, Nicolette Roelina den Braver, Karin Johanna Borgonjen-van den Berg, Femke Rutters, Amber van der Heijden, Joline Wilhelma Johanna Beulens

**Affiliations:** 1 Department of Epidemiology and Data Science, Amsterdam UMC, Vrije Universiteit Amsterdam, Amsterdam Public Health Research Institute, Amsterdam, The Netherlands; 2 Department of Agrotechnology and Food Sciences, Division of Human Nutrition and Health, Wageningen University and Research, Wageningen, The Netherlands; 3 Department of General Practice, Amsterdam UMC, Vrije Universiteit Amsterdam, Amsterdam Public Health Research Institute, Amsterdam, The Netherlands; 4 Julius Center for Health Sciences and Primary Care, University Medical Center Utrecht, Utrecht, The Netherlands

**Keywords:** Cardio-metabolic parameters, Dietary pattern, Dutch healthy diet index 2015, Glycemic control, Type 2 diabetes

## Abstract

**Purpose:**

To investigate whether adherence to the Dutch Healthy Diet index 2015 (DHD15-index) is associated with change in glycemic control and cardio-metabolic markers over two-year follow-up in people with type 2 diabetes (T2D).

**Methods:**

This prospective cohort study included 1202 individuals with T2D (mean age 68.7 ± 9.0 years; 62.5% male; mean HbA1c 53.8 ± 11.7 mmol/mol) from the Diabetes Care System cohort. Baseline dietary intake was assessed using a validated food frequency questionnaire, and adherence to the DHD15-index was estimated (range 0–130). HbA1c, fasting glucose, blood lipids (HDL and LDL cholesterol, cholesterol ratio), blood pressure, estimated glomerular filtration rate (eGFR), and BMI were measured at baseline, and after one- and two-year follow-up. Linear mixed model analyses were conducted to examine the associations between adherence to the DHD15-index and glycemic control and the cardio-metabolic outcomes, adjusting for energy intake, sociodemographic and lifestyle characteristics, and medication.

**Results:**

Highest adherence (T3) to the DHD15-index was not associated with change in HbA1c, compared to lowest adherence (T1) [β_T3vsT1_: 0.62 mmol/mol (− 0.94; 2.19), *P*_trend_ = 0.44]. There was a non-linear association with fasting glucose, where moderate adherence (T2) was associated with a decrease in fasting glucose [β_T2vsT1_: − 0.29 mmol/L (− 0.55; − 0.03), *P*_trend_ = 0.30]. Higher adherence to the DHD15-index was associated with a decrease in BMI [β_10point_: − 0.41 kg/m^2^ (− 0.60; − 0.21), *P*_trend_ < 0.001], but not with blood lipids, blood pressure or kidney function.

**Conclusion:**

In this well-controlled population of people with T2D, adherence to the DHD15-index was associated with a decrease in BMI, but not with change in glycemic control or other cardio-metabolic parameters.

**Supplementary Information:**

The online version contains supplementary material available at 10.1007/s00394-022-02847-6.

## Introduction

It is important to improve diabetes management to prevent comorbidities and complications among people with type 2 diabetes (T2D), such as cardiovascular disease, nephropathy, retinopathy and neuropathy [1]. Optimal glycemic control and cardio-metabolic health are therefore essential [2, 3], which can be achieved by lifestyle factors, including a healthy diet [1]. Studies have shown that adequate dietary management is associated with a reduction in hemoglobin A1c (HbA1c) levels of 11.0–22.0 mmol/mol [4].

Specifically, diets high in vegetables, fruits, legumes, nuts, poultry and vegetable oil, and low in solid fats, are associated with lower mortality rates in people with T2D [5], intake of low glycemic index fruits, nuts, whole grain products, and dairy is associated with improved glycemic control [6–9], and intake of low glycemic index fruits, nuts and wholegrain products with improved cardio-metabolic markers in people with T2D [6, 8, 10]. Systematic reviews of randomized controlled trials (RCTs) on dietary patterns, such as the Mediterranean diet score, have shown beneficial effects of the Mediterranean diet on glycemic control and cardio-metabolic markers in Western populations, reducing HbA1c by 1.1–6.6 mmol/mol [11–14], fasting glucose by 0.4–2.2 mmol/L [11, 13], low-density lipoprotein (LDL) cholesterol non-significantly by 0.08–0.19 mmol/L, increasing high-density lipoprotein (HDL) cholesterol by 0.04–0.09 mmol/L [11, 14, 15], and reducing systolic blood pressure (SBP) by 1.45 mm Hg, diastolic blood pressure (DBP) by 1.41 mm Hg, and body mass index (BMI) by 0.29 kg/m^2^ [11]. The Mediterranean diet however cannot directly be translated to the Dutch dietary behavior.

In 2015, the Dutch Health Council developed novel dietary guidelines largely consistent with the Mediterranean diet, focusing on food groups rather than nutrients, applicable to the Dutch general population and easy to understand and implement [16], aimed at facilitating adherence [17]. To evaluate adherence to the Dutch dietary guidelines, the Dutch Healthy Diet index of 2015 (DHD15-index) has been developed [18]. Higher adherence to the DHD15-index is associated with lower T2D incidence and lower all-cause mortality in the Netherlands [19, 20]. Dietary guidelines for people with T2D also mainly advocate a dietary pattern in line with the Dutch Healthy Diet guidelines and Mediterranean diet [21]. However, to date, no studies investigated the association between adherence to the DHD15-index and glycemic control or cardio-metabolic markers in people with T2D. Moreover, to our knowledge, no prior studies prospectively examined the association between dietary patterns and glycemic control or cardio-metabolic markers in people with T2D.

Therefore, the aim of this study was to investigate whether adherence to the DHD15-index was associated with change in glycemic control and cardio-metabolic markers over one- and two-year follow-up in people with T2D.

## Methods

### Study design and population

**Fig. 1:**
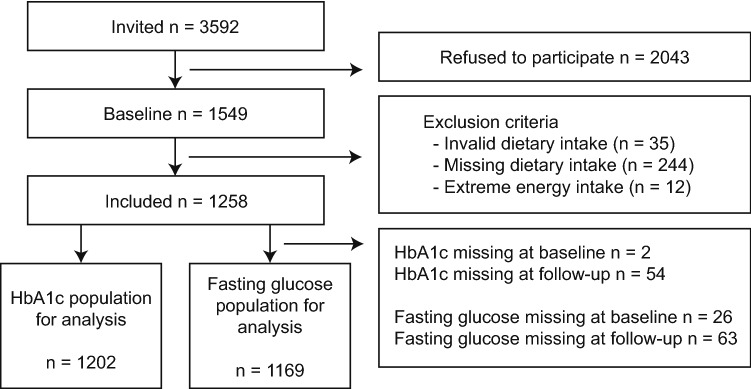
Flowchart study population recruitment and retention

This study was embedded in the Diabetes Care System cohort (DCS), a prospective dynamic patient cohort including nearly all people with T2D with GPs located in the West-Friesland region of the Netherlands [22], consisting of ~ 15,000 persons in 2020. Participants visit the care center annually for monitoring visits during which routine measurements are performed. Between June 2017 and June 2018, 3592 individuals were invited to participate in a sub-study, of which 1549 participated (43%) (Fig. 1). Inclusion criteria were T2D diagnosis based on either having at least one classic T2D symptom (polyuria, polydipsia, polyphagia, unintended weight loss, pruritus) together with elevated blood glucose levels (fasting glucose ≥ 7.0 mmol/L, or random glucose ≥ 11.1 mmol/L), or, in case of no symptoms, having two or more elevated blood glucose levels on two separate occasions [22], and ability to provide informed consent. Routine measurements data were collected from the medical records during the annual visit, and for the present study additional data on dietary intake, lifestyle, behavior and health were obtained using questionnaires, and data on physical activity using accelerometers. The study was approved by the Ethical Review Committee of the VU University Medical Center in Amsterdam. All participants provided written informed consent.

### Dietary assessment

Dietary intake was assessed at baseline using a self-reported 160-item food frequency questionnaire (FFQ): the FFQ-NL 1.0 [23]. This FFQ was specifically developed for Dutch observational studies, and validated against 24-h dietary recalls (on average 2.7 per person) and biomarkers in 24-h blood and urine. The FFQ was found to have a moderate validity in ranking individuals according to micronutrients, macronutrients and energy, and food groups, with median correlation coefficients of 0.30, 0.39 and 0.30, respectively [23]. The FFQ-NL 1.0 contains questions on frequency (times per month/week), portion size (standardized household measures) and preparation methods regarding 160 food items. The reference period for the FFQ was one year. Average daily intakes per food item (g/day) were derived and categorized into DHD15-index food groups.

### Dutch healthy diet index

The DHD15-index comprises fifteen components, divided over five component types: adequacy, moderation, optimum, ratio and quality [18]. An overview of the calculation methods, cut-off and threshold values per component is provided in Online Resource 1, details are described elsewhere [18].

The *adequacy components* are vegetables, fruits, wholegrain products, legumes, nuts, fish and tea. In this component, a higher intake means a higher score (better diet quality), and intakes beyond the prescribed cut-off value are assigned 10 points (maximum score). The score is calculated by dividing the intake by the cut-off value and multiplying it by 10. Adequacy score = $\frac{\mathrm{intake}}{\mathrm{cut}-\mathrm{off \: value}}*10$. The *moderation components* are red meat, processed meat, sugar-sweetened beverages, fruit juices, alcohol, and sodium, for which a lower intake means a higher score, and intakes below a prescribed threshold value are assigned 10 points. The score is calculated by taking the ratio between the intake minus the cut-off value and the threshold minus the cut-off value, multiplying it by 10 and subtracting it from 10. Moderation score = $10- \frac{\mathrm{intake }-\mathrm{ cut}-\mathrm{off \: value}}{\mathrm{threshold \: value }-\mathrm{ cut}-\mathrm{off \: value}} *10$. For the *optimum component*, including dairy products, intakes between a specified optimal range are assigned the highest score. The score for intakes below the lower cut-off value of the optimal range is calculated by dividing the intake by the lower cut-off value and multiplying this ratio by 10. Optimum score lower intakes = $\frac{\mathrm{intake}}{\mathrm{cut}-\mathrm{off \: value}} *10$. The score for intakes between the higher cut-off value of the optimal range and the threshold value is calculated by subtracting the cut-off value from the intake and dividing this by the difference between the threshold and cut-off value, multiplying it by 10 and subtracting it from 10. Optimum score higher intakes = $10- \frac{\mathrm{intake }-\mathrm{ cut}-\mathrm{off \: value}}{\mathrm{threshold \: value}-\mathrm{cut}-\mathrm{off \: value}} *10$. In the *ratio component*, containing fats/oils and refined/wholegrain products, a higher healthy/unhealthy ratio is assigned a higher score, and ratios above the specified cut-off value are assigned 10 points. The score is calculated by subtracting the threshold value from the healthy/unhealthy ratio, and dividing this by the difference between the cut-off and threshold value. Ratio score = $\frac{\mathrm{intake \: ratio }-\mathrm{ threshold \: value}}{\mathrm{cut}-\mathrm{off \: value}-\mathrm{ threshold \: value}}$. Since wholegrain products are included both as adequacy and ratio component, a score of 0 (minimum score) to 5 (maximum score) is assigned within each component, creating one total wholegrain products score. Lastly, in the *quality component*, comprising coffee, the recommended product within a particular type of product is assigned the highest score. In this case, no or filtered coffee consumption is assigned 10 points, and unfiltered coffee consumption 0 points. The FFQ used for the present study could not distinguish between unfiltered and filtered coffee and could not estimate sodium intake, these components were therefore excluded from the DHD15-index.

Each component received a score of 0–10 points. The final DHD15-index score is calculated by summing all components, generating scores ranging from 0 (lowest adherence) to 130 (highest adherence). For the analyses, the DHD15-index was used both as tertiles and continuously per 10 point increase.

### Outcome assessment

The primary outcomes were HbA1c and fasting glucose levels, measured at baseline and after one and two years of follow-up. Fasting blood samples were drawn during the annual monitoring visits, after an overnight fast. HbA1c levels (percentage and mmol/mol) were assessed through the turbidimetric inhibition immunoassay for hemolyzed whole EDTA blood (Cobas c501, Roche Diagnostics, Mannheim, Germany). Fasting glucose levels (mmol/L) were assessed in fluorinated plasma with the UV-hexokinase test (Cobas c501, Roche Diagnostics) [22].

Secondary outcomes included HDL cholesterol, LDL cholesterol, cholesterol ratio, SBP, DBP, eGFR, and BMI, also measured at baseline and after one and two years of follow-up. Both HDL and LDL cholesterol levels were derived from fasting blood samples, HDL cholesterol levels were established enzymatically (Cobas c501, Roche Diagnostics), and LDL cholesterol levels were calculated according to the formula: $LDL \,cholesterol=total \,cholesterol-HDL \,cholesterol-0.45*triglycerides$. The cholesterol ratio was calculated by dividing total cholesterol by HDL cholesterol. SBP and DBP were measured in duplicate per visit using an oscillometric device (Welch Allyn ProBP 3400, Skaneateles Falls, New York, USA), of which the averages were taken. The eGFR was measured using an overnight first morning urine sample, and calculated by means of the Modification of Diet in Renal Disease (MDRD) equation [24]. BMI was calculated by dividing body weight (kilograms) by height (meters) squared, measured barefoot and in light clothing.

### Covariates

Sociodemographic characteristics were retrieved from self-reported general questionnaires. Age was determined based on date of birth (baseline and follow-up). Diabetes duration was calculated from medical records, by subtracting the diagnosis date from the annual visit date. Self-reported education was categorized into low (no completed education, primary education, secondary education (practical training)), middle (pre-vocational secondary education, vocational training, general secondary education or pre-university education), or high (professional university education, university). Employment status was categorized into employed (paid work, volunteer work) or unemployed/retired (unemployed, retired). Smoking status was categorized as current, former or never smoker. Physical activity was measured at baseline using accelerometers (ActiGraph GT3X, worn on the hip for 8 days) and reported as total hours of physical activity per week. Glucose-lowering medication was obtained from medication labels during the annual visits (baseline and follow-up), and categorized into no medication, one oral hypoglycemic agent (ATC code A10B), ≥ two oral hypoglycemic agents, or insulin (ATC code A10A)/combination therapy (oral hypoglycemic agents + insulin). Lipid-lowering and antihypertensive medication were also retrieved from medication labels during the visits and were dichotomous (no medication, medication).

### Statistical analysis

Baseline characteristics were presented as mean ± SD, median (IQR), or proportions (*n* (%)), as appropriate based on unit and normality, for the total population and by tertiles of DHD15 adherence at baseline. Missing data in covariates (30.3% of participants had missing values for one or more covariates, ranging from 0.2% missing for smoking status and BMI, to 24.7% for physical activity) were imputed using multiple imputation, with five sets of imputed data based on predictive mean matching, and pooled according to Rubin’s rules [25].

Separate linear mixed models for repeated measures were used to examine the association between adherence to the DHD15-index at baseline and change in HbA1c (%), HbA1c (mmol/mol), fasting glucose, HDL cholesterol, LDL cholesterol, cholesterol ratio, SBP, DBP, eGFR and BMI over two years of follow-up. Unstandardized regression coefficients and 95% confidence intervals were presented per tertile (lowest tertile reference), representing the change in cardio-metabolic parameter over two years of follow-up from the moderate and highest tertile of adherence relative to the lowest tertile, and per 10 point increase in adherence to the index. Linearity across tertiles was assessed by adding the categorical DHD15-index variable as a linear term to the model. Non-linearity was explored by adding a quadratic DHD15-index term, which was not significant in any of the models.

For each outcome, four models were created (except for eGFR, where three models were created, and BMI, where two models were created). The first model was adjusted for age, sex and total energy intake. The second model was additionally adjusted for education, employment status, smoking, and physical activity. BMI was additionally added in a separate model (model 3) because of its potential role as mediator [26]. Finally, a fourth model was constructed where, depending on the outcome, glucose-lowering, lipid-lowering or antihypertensive medication was added to model 2, because these medications strongly affect glycemic control [27], blood lipids [28] and blood pressure [29], respectively. Confounders were added based on theory and previous literature. Age, sex, current smoking status and diabetes duration were checked for effect modification in model 2 by testing interaction. In case the interaction term was significant (*p* < 0.05), analyses were stratified. Each model included adherence to the DHD15-index at baseline and the confounding variables as fixed effects, whereas participant ID was treated as a random effect to account for potential within-participant correlations among the repeated measures of the cardio-metabolic parameter over follow-up. Likelihood ratio tests were conducted to compare model fit of the model including a random intercept on participant ID with the model additionally including a random slope. In case of significant improvement in model fit, the model including the random slope was used for analyses. To control the type I error rate, multiple testing was statistically accounted for using the post hoc Bonferroni correction [30].

We conducted a sensitivity analysis comparing baseline characteristics of the excluded participants with those of the included participants, to evaluate whether selection bias occurred. Moreover, complete case analyses including participants with complete data on all covariates and restricting to participants with both follow-up measurements, were conducted as sensitivity analyses, to assess the impact of data imputation and missing follow-up data. Additionally, we performed a sensitivity analysis excluding potential energy under-reporters (those with a reported energy intake to basal metabolic rate ratio below the lower confidence interval limit corresponding to their physical activity level, ranging from 0.68 to 1.24) according to the Goldberg method [31–33], to evaluate whether desire for social approval caused underreporting of discretionary foods, causing measurement error and differential misclassification, leading to underestimations. Lastly, sensitivity analyses excluding alcohol from the DHD15-index were conducted, to assess the impact of potential misclassification in the scoring of alcohol intake conforming to the DHD15-index, as this scoring is not entirely in line with previous studies observing a dose–response relationship between alcohol consumption and HDL cholesterol [34], and a J-shaped association between alcohol consumption and glycemic control [35] and eGFR decline [36, 37].

To evaluate associations, a significance level of *p* < 0.05 was used (*p* < 0.0045 after Bonferroni correction based on 11 comparisons). All statistical analyses were conducted using SPSS Statistics 26.

## Results

For the current study, we excluded participants with invalid (*n* = 35) or missing (*n* = 244) data on dietary intake, or with extreme energy intake (top and bottom 0.5%) (*n* = 12). In addition, we excluded participants with missing HbA1c (*n* = 56) or fasting glucose (*n* = 89) data at baseline or both follow-up measurements (Fig. 1). The same procedure was applied for missing data in the secondary outcomes: HDL cholesterol (*n* = 56), LDL cholesterol (*n* = 57), cholesterol ratio (*n* = 56), SBP (*n* = 54), DBP (*n* = 54), estimated glomerular filtration rate (eGFR) (*n* = 55), and BMI (*n* = 56).

### Baseline characteristics

Baseline characteristics in total and stratified by DHD15-index tertiles are presented in Table 1. The participants were on average 68.7 ± 9.0 years old, 62.5% were male. The mean score for adherence to the DHD15-index at baseline was 72.0 ± 14.9. Adherence was highest for red meat and legume guidelines (respectively 75.0% and 74.5% full adherence), and lowest for processed meat and grain guidelines (respectively 1.2% and 3.3% full adherence). None of the participants fully adhered to all guidelines. Highest adherers (T3; score 78.4–116.1) were more often female, higher educated, employed, non-smoking, more physically active, and had a lower BMI and total energy intake, compared to lowest adherers (T1; score 21.0–65.6). Participants had a mean HbA1c level of 53.8 ± 11.7 mmol/mol at baseline, with a mean change of 3.4 ± 11.1 after two years of follow-up, and a mean fasting glucose level of 8.6 ± 2.1 mmol/L at baseline, with a mean change of 0.7 ± 2.5, indicating that the participants are well-controlled (Table 2). There were no major differences in baseline values across the tertiles. Baseline characteristics of the excluded participants did not differ substantially from those of the included participants (Online Resource 2).

**Table 1:** Baseline characteristics (*n* = 1202) presented as mean ± SD, median (IQR), or *n* (%)

Participant characteristic	Total population	DHD15-index tertiles		
		T1 (*n* = 400)	T2 (*n* = 401)	T3 (*n* = 401)
		< 65.6	65.6 – 78.4	> 78.4
Sex (male)	751 (62.5%)	292 (73.0%)	269 (67.1%)	190 (47.4%)
Age (years)	68.7 ± 9.0	68.9 ± 9.3	68.5 ± 9.0	68.8 ± 8.8
Diabetes duration (years)	12.8 ± 5.9	12.7 ± 5.9	12.8 ± 6.1	12.8 ± 5.7
Education				
Low Middle High	356 (30.1%) 565 (47.8%) 260 (22.0%)	134 (34.0%) 179 (45.4%) 81 (20.6%)	112 (28.4%) 191 (48.4%) 92 (23.3%)	111 (28.3%) 195 (49.7%) 86 (21.9%)
Employment status (employed)	362 (31.8%)	115 (30.3%)	113 (29.6%)	134 (35.4%)
Smoking				
Current Former Never	123 (10.3%) 683 (56.9%) 394 (32.8%)	66 (16.5%) 233 (58.3%) 101 (25.3%)	39 (9.8%) 243 (60.9%) 117 (29.3%)	19 (4.7%) 207 (51.6%) 175 (43.6%)
Physical activity (hours/week)	1.4 (2.3)	1.0 (2.0)	1.5 (2.5)	1.6 (2.2)
Glucose-lowering medication				
No medication One OHA ≥ Two OHA Only insulin OHA + insulin	199 (16.6%) 356 (29.6%) 286 (23.8%) 59 (4.9%) 302 (25.1%)	63 (15.8%) 115 (28.7%) 100 (25.0%) 23 (5.8%) 99 (24.7%)	71 (17.7%) 117 (29.2%) 90 (22.4%) 15 (3.7%) 108 (26.9%)	64 (16.0%) 125 (31.2%) 96 (23.9%) 21 (5.2%) 95 (23.7%)
Total energy intake (kcal/day)	2140 ± 733	2147 ± 764	2158 ± 739	2119 ± 693
DHD15-index score	72.0 ± 14.9	55.6 ± 7.9	72.2 ± 3.7	88.2 ± 7.4
DHD15 components (g/day)				
Fruits Vegetables Wholegrain Refined grain Legumes Nuts Cheese Dairy Lean fish Fatty fish Tea Liquid fat Solid fat Red meat Processed meat SSB Alcohol	124.6 (170.1) 102.8 (106.4) 70.5 (113.2) 82.2 (104.7) 25.4 (41.1) 5.5 (11.2) 22.3 (29.0) 150.0 (190.7) 10.5 (14.4) 7.3 (12.8) 170.0 (341.7) 19.6 (24.2) 14.0 (26.7) 23.3 (34.5) 51.6 (43.1) 86.6 (240.3) 3.8 (16.9)	59.1 (111.8) 68.5 (82.0) 32.1 (93.2) 104.6 (115.4) 16.3 (39.6) 1.7 (5.6) 21.3 (24.6) 104.9 (156.9) 7.1 (14.8) 4.3 (9.5) 30.6 (170.0) 18.8 (27.2) 17.8 (28.7) 27.0 (43.8) 57.5 (45.8) 175.0 (325.0) 7.7 (25.3)	122.7 (152.4) 105.7 (102.1) 70.5 (99.3) 83.4 (105.2) 25.4 (41.7) 5.6 (11.2) 21.7 (29.7) 150.0 (181.1) 10.9 (13.7) 6.4 (11.9) 170.0 (309.4) 19.6 (22.3) 16.4 (26.5) 23.8 (34.9) 53.9 (42.1) 124.3 (243.0) 4.5 (16.7)	219.1 (118.2) 133.5 (114.3) 91.8 (95.6) 67.8 (79.2) 28.1 (39.7) 9.4 (14.6) 25.2 (29.2) 191.6 (189.1) 12.9 (15.0) 11.8 (13.7) 340.0 (365.5) 21.2 (23.9) 7.1 (22.0) 21.1 (30.4) 44.6 (42.9) 40.7 (105.2) 2.3 (8.1)
HbA1c (mmol/mol)	53.8 ± 11.7	54.1 ± 11.4	53.1 ± 11.7	54.3 ± 12.0
Fasting glucose (mmol/L)	8.6 ± 2.1	8.8 ± 2.1	8.4 ± 2.1	8.6 ± 2.2
HDL cholesterol (mmol/L), women	1.4 ± 0.4	1.3 ± 0.4	1.4 ± 0.4	1.5 ± 0.4
HDL cholesterol (mmol/L), men	1.2 ± 0.3	1.2 ± 0.4	1.2 ± 0.3	1.2 ± 0.3
LDL cholesterol (mmol/L)	2.2 ± 0.9	2.1 ± 0.9	2.2 ± 0.8	2.2 ± 0.9
Cholesterol ratio	3.3 (1.5)	3.5 (1.4)	3.4 (1.6)	3.1 (1.4)
Systolic blood pressure (mm Hg)	140.7 ± 20.3	142.3 ± 19.6	139.3 ± 20.1	140.3 ± 21.0
Diastolic blood pressure (mm Hg)	77.8 ± 8.0	78.1 ± 8.3	77.7 ± 8.4	77.5 ± 7.2
eGFR (ml/min)	73.9 ± 18.2	73.8 ± 19.1	73.3 ± 17.9	74.4 ± 17.4
BMI (kg/m^2^)	29.6 ± 5.2	30.2 ± 5.4	29.5 ± 4.9	29.2 ± 5.2

*OHA* oral hypoglycemic agents, *DHD15* Dutch Healthy Diet index 2015, *SSB* sugar-sweetened beverages, *HbA1c* hemoglobin A1c, *LDL* low-density lipoprotein, *HDL* high-density lipoprotein, *eGFR* estimated glomerular filtration rate, *BMI* body mass index

**Table 2:** Changes in cardio-metabolic parameters after two years of follow-up presented as mean ± SD (*n* = 921)

Cardio-metabolic parameter	Total population	DHD15-index tertiles		
		T1 (*n* = 400)	T2 (*n* = 401)	T3 (*n* = 401)
		< 65.6	65.6–78.4	> 78.4
Δ HbA1c (mmol/mol)	3.35 ± 11.14	2.87 ± 12.18	3.52 ± 10.33	3.65 ± 10.89
Δ Fasting glucose (mmol/L)	0.67 ± 2.54	0.59 ± 2.88	0.64 ± 2.30	0.77 ± 2.40
Δ HDL cholesterol (mmol/L), women	0.00 ± 0.21	0.00 ± 0.21	0.02 ± 0.22	− 0.01 ± 0.20
Δ HDL cholesterol (mmol/L), men	0.00 ± 0.19	− 0.02 ± 0.21	− 0.01 ± 0.16	0.02 ± 0.20
Δ LDL cholesterol (mmol/L)	− 0.08 ± 0.72	− 0.10 ± 0.74	− 0.05 ± 0.75	− 0.08 ± 0.67
Δ Cholesterol ratio	− 0.08 ± 0.90	− 0.04 ± 0.92	− 0.08 ± 0.93	− 0.11 ± 0.87
Δ Systolic blood pressure (mm Hg)	0.81 ± 17.32	− 0.10 ± 16.41	1.10 ± 17.26	1.53 ± 18.21
Δ Diastolic blood pressure (mm Hg)	− 0.78 ± 6.94	− 0.99 ± 7.14	− 0.58 ± 7.10	− 0.76 ± 6.55
Δ eGFR (ml/min)	4.72 ± 10.30	3.79 ± 11.04	4.40 ± 9.44	5.99 ± 10.33
Δ BMI (kg/m^2^)	− 0.32 ± 1.59	− 0.39 ± 1.65	− 0.21 ± 1.41	− 0.39 ± 1.70

*DHD15* Dutch Healthy Diet index 2015, *HbA1c* hemoglobin A1c, *LDL* low-density lipoprotein, *HDL* high-density lipoprotein, *eGFR* estimated glomerular filtration rate, *BMI* body mass index, *Δ* change in cardio-metabolic parameter after 2 years of follow-up

### Primary outcomes

No random slopes were included in any of the primary models. In the model adjusted for age, sex, and total energy intake, highest adherence (T3) to the DHD15-index at baseline was not associated with change in HbA1c, compared to lowest adherence (T1) (model 1; T3vsT1: β = 0.37 mmol/mol [95% CI = − 1.16; 1.90]), as was the case for the fully adjusted model (model 2; T3vsT1: β = 0.62 mmol/mol [95% CI = − 0.94; 2.19]) (Table 3). No significant linear trend was found (*P*_trend_ = 0.44). Additional adjustment for BMI amplified the association (model 3; T3vsT1: β = 1.10 mmol/mol [95% CI = − 0.47; 2.68]), whereas additional adjustment for glucose-lowering medication attenuated the association slightly (model 4; T3vsT1: β = 0.58 mmol/mol [95% CI = − 0.76; 1.92]). For comprehensibility, Online Resource 3 contains the HbA1c results in percentage units.

**Table 3:** Association between adherence to the DHD15-index at baseline and change in cardio-metabolic parameters (*n* = 1202) ^a^

HbA1c (mmol/mol)	T1	T2		T3		*P* for trend	Continuous (per 10 point)	
		β	95% CI	β	95% CI		β	95% CI
Model 1	Ref.	− 0.46	− 1.95; 1.03	0.37	− 1.16; 1.90	0.65	0.08	− 0.34; 0.50
Model 2	Ref.	− 0.11	− 1.61; 1.39	0.62	− 0.94; 2.19	0.44	0.17	− 0.27; 0.61
Model 3	Ref.	0.13	− 1.37; 1.63	1.10	− 0.47; 2.68	0.17	0.33	− 0.12; 0.77
Model 4	Ref.	− 0.10	− 1.38; 1.18	0.58	− 0.76; 1.92	0.40	0.17	− 0.21; 0.54

Fasting glucose (mmol/L)	T1	T2		T3		*P* for trend	Continuous (per 10 point)	
		β	95% CI	β	95% CI		β	95% CI
Model 1	Ref.	− 0.33	− 0.59; − 0.07*	− 0.14	− 0.40; 0.12	0.29	− 0.05	− 0.12; 0.02
Model 2	Ref.	− 0.29	− 0.55; − 0.03*	− 0.14	− 0.41; 0.13	0.30	− 0.05	− 0.13; 0.03
Model 3	Ref.	− 0.28	− 0.54; − 0.02*	− 0.09	− 0.36; 0.18	0.49	− 0.04	− 0.11; 0.04
Model 4	Ref.	− 0.26	− 0.50; − 0.01*	− 0.14	− 0.39; 0.11	0.27	− 0.04	− 0.11; 0.03

HDL cholesterol (mmol/L), women	T1	T2		T3		*P* for trend	Continuous (per 10 point)	
		β	95% CI	β	95% CI		β	95% CI
Model 1	Ref.	0.03	− 0.07; 0.12	0.13	0.04; 0.22**	0.002**	0.04	0.01; 0.06**
Model 2	Ref.	0.00	− 0.10; 0.09	0.08	− 0.01; 0.17	0.06	0.02	0.00; 0.05
Model 3	Ref.	− 0.02	− 0.11; 0.08	0.06	− 0.03; 0.15	0.11	0.02	− 0.01; 0.04
Model 4	Ref.	0.00	− 0.10; 0.09	0.08	− 0.01; 0.17	0.06	0.02	0.00; 0.05

HDL cholesterol (mmol/L), men	T1	T2		T3		*P* for trend	Continuous (per 10 point)	
		β	95% CI	β	95% CI		β	95% CI
Model 1	Ref.	− 0.01	− 0.06; 0.04	0.01	− 0.05; 0.07	0.80	0.00	− 0.02; 0.02
Model 2	Ref.	− 0.03	− 0.08; 0.02	− 0.01	− 0.07; 0.04	0.58	− 0.01	− 0.03; 0.01
Model 3	Ref.	− 0.03	− 0.08; 0.02	− 0.04	− 0.09; 0.02	0.18	− 0.02	− 0.03; 0.00*
Model 4	Ref.	− 0.03	− 0.08; 0.02	− 0.01	− 0.07; 0.04	0.58	− 0.01	− 0.03; 0.01

LDL cholesterol (mmol/L)	T1	T2		T3		*P* for trend	Continuous (per 10 point)	
		β	95% CI	β	95% CI		β	95% CI
Model 1	Ref.	0.04	− 0.06; 0.15	0.05	− 0.06; 0.16	0.37	0.03	0.00; 0.06
Model 2	Ref.	0.03	− 0.08; 0.14	0.04	− 0.08; 0.15	0.54	0.02	− 0.01; 0.06
Model 3	Ref.	0.03	− 0.08; 0.14	0.04	− 0.08; 0.15	0.51	0.03	− 0.01; 0.06
Model 4	Ref.	0.03	− 0.08; 0.13	0.03	− 0.08; 0.14	0.59	0.02	− 0.01; 0.05

Cholesterol ratio	T1	T2		T3		*P* for trend	Continuous (per 10 point)	
		β	95% CI	β	95% CI		β	95% CI
Model 1	Ref.	0.04	− 0.11; 0.19	− 0.13	− 0.29; 0.02	0.10	− 0.03	− 0.07; 0.02
Model 2	Ref.	0.08	− 0.07; 0.23	− 0.06	− 0.22; 0.10	0.44	− 0.01	− 0.05; 0.04
Model 3	Ref.	0.10	− 0.05; 0.25	− 0.02	− 0.18; 0.14	0.81	0.01	− 0.04; 0.05
Model 4	Ref.	0.08	− 0.07; 0.23	− 0.07	− 0.22; 0.09	0.41	− 0.01	− 0.05; 0.04

SBP (mm Hg)	T1	T2		T3		*P* for trend	Continuous (per 10 point)	
		β	95% CI	β	95% CI		β	95% CI
Model 1	Ref.	− 2.09	− 4.41; 0.22	− 1.26	− 3.63; 1.11	0.29	− 0.37	− 1.02; 0.29
Model 2	Ref.	− 1.93	− 4.27; 0.40	− 1.17	− 3.60; 1.25	0.34	− 0.34	− 1.02; 0.34
Model 3	Ref.	− 1.53	− 3.87; 0.81	− 0.55	− 3.00; 1.89	0.65	− 0.18	− 0.86; 0.51
Model 4	Ref.	− 1.89	− 4.21; 0.44	− 1.10	− 3.52; 1.32	0.36	− 0.32	− 1.00; 0.35

DBP (mm Hg)	T1	T2		T3		*P* for trend	Continuous (per 10 point)	
		β	95% CI	β	95% CI		β	95% CI
Model 1^b^	Ref.	− 0.24	− 1.20; 0.71	− 0.32	− 1.29; 0.65	0.52	− 0.05	− 0.31; 0.22
Model 2^b^	Ref.	− 0.41	− 1.36; 0.55	− 0.65	− 1.64; 0.34	0.20	− 0.17	− 0.44; 0.11
Model 3^b^	Ref.	− 0.13	− 1.07; 0.82	− 0.16	− 1.14; 0.83	0.77	− 0.02	− 0.29; 0.25
Model 4^b^	Ref.	− 0.40	− 1.36; 0.55	− 0.64	− 1.63; 0.35	0.21	− 0.16	− 0.44; 0.11

eGFR (ml/min)	T1	T2		T3		*P* for trend	Continuous (per 10 point)	
		β	95% CI	β	95% CI		β	95% CI
Model 1	Ref.	0.02	− 2.37; 2.41	1.95	− 0.50; 4.40	0.12	0.43	− 0.25; 1.11
Model 2	Ref.	0.11	− 2.30; 2.52	1.74	− 0.76; 4.25	0.18	0.40	− 0.30; 1.11
Model 3	Ref.	− 0.08	− 2.49; 2.33	1.42	− 1.09; 3.93	0.27	0.30	− 0.41; 1.00

BMI (kg/m^2^)	T1	T2		T3		*P* for trend	Continuous (per 10 point)	
		β	95% CI	β	95% CI		β	95% CI
Model 1	Ref.	− 0.83	− 1.51; − 0.15*	− 1.56	− 2.25; − 0.86**	< 0.001**	− 0.46	− 0.65; − 0.26**
Model 2	Ref.	− 0.69	− 1.37; − 0.01*	− 1.37	− 2.07; − 0.66**	< 0.001**	− 0.41	− 0.60; − 0.21**

Model 1: Adjusted for age, sex and total energy intake. Model 2: Additionally adjusted for education, employment status, smoking and physical activity. Model 3: Additionally adjusted for body mass index. Model 4: Model 2 additionally adjusted for glucose-lowering, lipid-lowering or antihypertensive medication

*β* unstandardized regression coefficient, *CI* confidence interval, *HbA1c* hemoglobin A1c, *LDL* low-density lipoprotein, *HDL* high-density lipoprotein, *SBP* systolic blood pressure, *DBP* diastolic blood pressure, *eGFR* estimated glomerular filtration rate, *BMI* body mass index

*p value < 0.05

**p value < Bonferroni-corrected alpha (= 0.0045)

^a^Participant ID included as random intercept

^b^Sex included as random slope

For changes in fasting glucose in all models, moderate adherence showed a significant decrease in fasting glucose, compared to lowest adherence (model 2; T2vsT1: β = − 0.29 mmol/L [95% CI = − 0.55; − 0.03]), and highest adherence pointed in the same direction (model 2; T3vsT1: β = − 0.14 mmol/L [95% CI = − 0.41; 0.13]), albeit not significant. No significant linear trend was observed (*P*_trend_ = 0.30). Additional adjustment for BMI slightly attenuated the association (model 3; T3vsT1: β = − 0.09 mmol/L [95% CI = − 0.36; 0.18]), while additional adjustment for glucose-lowering medication did not alter the association (model 4; T3vsT1: β = − 0.14 mmol/L [95% CI = − 0.39; 0.11]).

The complete case analyses (*n* = 608) (Online Resource 4), the analyses excluding under-reporters (excluding *n* = 123) (Online Resource 5), and the analyses excluding alcohol from the DHD15-index (*n* = 1202) (Online Resource 6) all yielded similar results to the main analyses. Bonferroni correction for multiple testing did not lead to different conclusions.

### Cardio-metabolic outcomes

A random slope on sex was included in the DBP analyses. Significant effect modification by sex was found for the HDL cholesterol analyses (*p* = 0.03). Stratification by sex showed that, although not significant, directionality of the association was different for men and women (Table 3). In men, highest adherence to the DHD15-index at baseline pointed toward a decrease in HDL cholesterol, compared to lowest adherence (model 2; T3vsT1: β = − 0.01 mmol/L [95% CI = − 0.07; 0.04], *P*_trend_ = 0.58), while among women the association pointed toward an increase in HDL cholesterol (model 2; T3 vs T1: β = 0.08 mmol/L [95% CI = − 0.01; 0.17], *P*_trend_ = 0.06). Higher adherence was not associated with changes in LDL cholesterol (model 2; T3vsT1: β = 0.04 mmol/L [95% CI = − 0.08; 0.15], *P*_trend_ = 0.54), or the cholesterol ratio (model 2; T3 vs T1: β = − 0.06 [95% CI = − 0.22; 0.10], *P*_trend_ = 0.44). In addition, no association was observed between higher adherence to the DHD15-index at baseline and SBP (model 2; T3vsT1: β = − 1.17 mm Hg [95% CI = − 3.60; 1.25], P_trend_ = 0.34), or DBP (model 2; T3vsT1: β = − 0.65 mm Hg [95% CI = − 1.64; 0.34], *P*_trend_ = 0.20). Higher adherence also was not associated with changes in eGFR (model 2; T3vsT1: β = 1.74 ml/min [95% CI = − 0.76; 4.25], *P*_trend_ = 0.18). Finally, higher adherence to the DHD15-index at baseline was associated with a decrease in BMI (model 2; 10 point: β = − 0.41 kg/m^2^ [95% CI = − 0.60; − 0.21], *P*_trend_ < 0.001).

The complete case analyses (Online Resource 4), the analyses excluding under-reporters (Online Resource 5), and the analyses excluding alcohol from the DHD15-index (Online Resource 6) all showed similar results to the main analyses. Application of the Bonferroni correction did not alter the results.

## Discussion

This study aimed to investigate the association between adherence to the DHD15-index at baseline and change in glycemic control and cardio-metabolic markers over one and two years of follow-up in people with T2D being well-controlled (medically monitored) in the Diabetes Care System. Higher adherence to the DHD15-index was associated with a lower BMI, and with a higher HDL cholesterol only in women. There was no association between adherence and changes in HbA1c, FPG, LDL cholesterol, cholesterol ratio, SBP, DBP or eGFR.

Our results regarding glycemic control seem to contradict RCTs investigating the effect of adherence to the Mediterranean diet, which is comparable to the DHD15-index, on cardio-metabolic parameters in people with T2D. These studies showed that adherence to the Mediterranean diet reduced HbA1c by 1.1–6.6 mmol/mol [11–14], and fasting glucose by 0.4–2.2 mmol/L [11, 13], after follow-up periods ranging from four weeks to four years. Our study observed no association between highest adherence to the DHD15-index at baseline and change in HbA1c or fasting glucose, compared to lowest adherence. The observational design of our study may have limited us from finding an association, as opposed to these previous RCTs. RCTs implement interventions in controlled settings, leading to stronger effects in shorter time periods than observational studies conducted in real-life settings. Regarding previous observational studies, one cross-sectional study observed no association between adherence to the Mediterranean diet and odds of having a high HbA1c level (≥ 53 mmol/mol) [38], similar to our findings, while two cross-sectional studies observed a significant association between a high adherence to the Mediterranean diet and respectively 1.1 and 9.9 mmol/mol lower mean HbA1c levels, compared to a low adherence [39, 40]. An explanation for these inconsistent findings among previous cross-sectional studies could be that with a mean age of 69 years versus 62 and 58 years, respectively, and mean HbA1c levels of 52 mmol/mol versus 61 and 68 mmol/mol, participants in the former cross-sectional study were better controlled than those in the latter two studies and thereby showed weaker associations. The deviation in results between our study and the latter two cross-sectional studies may be explained by the study design. Within-person changes in longitudinal studies are more subtle than between-persons comparisons in cross-sectional designs. Furthermore, the discrepancies in food processing and preparation methods between the Netherlands and the countries where the previous studies were conducted (i.e., Spain, Italy, Greece, Israel, the United States, and Australia), and the differences between the Dutch dietary guidelines and the Mediterranean diet, may cause a distorted view in this comparison [41]. Finally, and most importantly, our study population generally had diabetes for a long time (average of 12.8 ± 5.9 years at baseline) and were well-controlled in the Diabetes Care System. Therefore, they may have been less likely to have variations in their glycemic control and had less room for improvement.

For the secondary outcomes, we only observed a significant association between higher adherence to the DHD15-index at baseline and a 0.41 kg/m^2^ decrease in BMI. This finding is in line with prior RCTs observing that adherence to the Mediterranean diet reduced BMI by 0.29 kg/m^2^ [11]. We expected to observe beneficial impacts of a higher adherence to the DHD15-index at baseline on cholesterol levels and blood pressure based on previous RCTs indicating that adherence to the Mediterranean diet non-significantly decreased LDL cholesterol by 0.08–0.19 mmol/L, and significantly increased HDL cholesterol by 0.04–0.09 mmol/L [11, 14, 15], and decreased SBP by 1.45 mm Hg and DBP by 1.41 mm Hg [11], in individuals with T2D. In our study, we observed no association between adherence to the DHD15-index at baseline and LDL cholesterol, SBP or DBP, although higher adherence was associated with higher HDL cholesterol, but only among women. A potential explanation for this deviation could, just as for our glycemic control results, lie within the observational longitudinal study design with only subtle changes in cardio-metabolic outcomes over time, that may require a longer time period to translate into significant improvement in cardiometabolic risk factors. Additionally, the observed association with HDL in women and close to significant association with HDL in men could be explained by the loss of statistical power after stratifying the study population. Moreover, we expected to observe a positive association between adherence to the DHD15-index and eGFR. One previous 15-year observational study concluded that a one point increase in Mediterranean diet score was associated with a 25% lower odds of rapid eGFR decline in participants without T2D after seven years of follow-up, but observed no association in participants with T2D [42]. Another two-year RCT found that adherence to the Mediterranean diet increased eGFR by 4.5% in participants without diabetes and by 6.7% in participants with diabetes, after two-year compliance [43]. These results are similar to ours: a non-significant 6.0 ml/min (8.1%) increase in eGFR in individuals with T2D in the highest tertile of adherence to the DHD15-index after two years of follow-up.

Strengths of the current study include the prospective two-year design with annual measurements, enabling the study to investigate temporality between exposure and outcome, the use of a population-based patient cohort, increasing the generalizability of the results to other populations with T2D, and the ability to adjust for many confounders. Moreover, contrary to previous studies, the present study was conducted two years after the implementation of the novel Dutch dietary guidelines in 2015. Participants therefore had the possibility to be aware of the guidelines and to adjust their diet accordingly. Nevertheless, some limitations should be considered. One limitation is that the cohort mainly consisted of Western European participants, and therefore the results cannot directly be generalized to other populations. Additionally, as in any observational study, our study could have suffered from (residual) confounding [44]. Furthermore, the FFQ was not specifically designed to measure DHD15-index food groups, which prevented (accurate) measurement of coffee and sodium, and led to the exclusion of these food groups from the DHD15-index, possibly affecting the results. However, although unfiltered coffee consumption, contrary to filtered coffee consumption, elevates total and LDL cholesterol levels [45], most coffee consumed in the Netherlands is filtered [46], indicating that there would be little variation among participants and therefore little impact on the results. Exclusion of sodium may have affected the SBP and DBP results, as excessive sodium intake is associated with increased blood pressure [47]. However, adding sodium intake to the FFQ probably would not have increased the validity of the results because FFQs are not accurate to estimate sodium intake, as added salt at the table or cooking cannot be accurately self-reported. Specifically, a previous study pooling data from five validation studies showed that the correlation coefficient for the correlation of sodium intake measured by an FFQ with true intake was only 0.16, and that the average underreporting of sodium measured by FFQs was 28–39%, leading to underestimations [48]. Another study on the previous DHD-index representing adherence to the Dutch Healthy Diet guidelines of 2006, showed a mean sodium component score of 2.4 when the score was estimated using 24-h urine samples, a score of 3.5 when using 24-h recalls, and a score of 4.8 when using FFQs, indicating substantial underestimations of sodium intake measured by 24-h recalls and FFQs within the DHD-index [49]. Finally, participants completed the FFQ only at baseline, while dietary intake may have changed over the two years of follow-up, which might lead to misclassification. Nevertheless, a prior study investigating the reproducibility of a validated FFQ in the European Investigation into Cancer and Nutrition (EPIC)-Heidelberg cohort concluded that with 60–70% of the participants being re-assigned to the same/adjacent food group intake quintile after 5.7 years of follow-up, food group intake is reasonably stable over time [50]. Since the time period of our study is shorter with only two years of follow-up, the misclassification is likely to be lower than in the EPIC-Heidelberg study. However, the EPIC-Heidelberg cohort recruited participants from the general population as opposed to from diabetes centers [51]. Dietary habits may differ between general populations and populations of people with T2D [52], and therefore caution should be taken when generalizing these results to our study population. Nonetheless, with the T2D duration among our participants ranging from 5.0 to 50.2 years at baseline, none of our participants were at the very beginning of medical treatment. Hence, it is likely that our participants had already adjusted and stabilized their diets to match the dietary prescriptions before they participated in our study, and that their diets did not alter greatly during the two years of follow-up.

In future research, larger-scale, prospective population-based studies with longer follow-up periods evaluating the association between adherence to dietary patterns and glycemic control in people with T2D are warranted. Evidence resulting from these studies may contribute to creating future dietary guidelines and improve dietary practices and disease management in individuals with T2D. Moreover, future Dutch dietary guidelines should additionally consider the effects of the guidelines on renal function, as chronic kidney disease is a serious public health burden associated with increased risks of kidney failure, cardiovascular disease, poor quality of life and mortality [53, 54], which a healthy diet may help prevent [55]. We conclude that there is no motive to advise individuals with T2D to deviate from the Dutch dietary guidelines.

In conclusion, in this population of people with T2D being well-controlled (medically monitored) in the Diabetes Care System, higher adherence to the DHD15-index at baseline was associated with a decrease in BMI over two years. Despite subtle changes in cardio-metabolic risk factors, this did not translate into associations with change in glycemic control or other cardio-metabolic risk factors.

## Supplementary Information

Below is the link to the electronic supplementary material.

Supplementary file1 (PDF 241 KB)

Supplementary file2 (PDF 497 KB)

Supplementary file3 (PDF 625 KB)

Supplementary file4 (PDF 621 KB)

Supplementary file5 (PDF 621 KB)

Supplementary file6 (PDF 618 KB)

## Data Availability

Not applicable.
