# Supplementary material for: Adherence to the Dutch healthy diet index and change in glycemic control and cardiometabolic markers in people with type 2 diabetes
Source: Eur J Nutr. 2022 Mar 14;61(5):2761–73. doi: 10.1007/s00394-022-02847-6 (PMC9279194; doi:10.1007/s00394-022-02847-6)
Supplement: Supplementary file 1 — Supplementary file1 (PDF 241 KB) [file 394_2022_2847_MOESM1_ESM.pdf]

Adherence to the Dutch Healthy Diet index and change in glycemic control and cardiometabolic markers in people with type 2 diabetes

Ehlana Catharina Maria Bartels[1], Nicolette Roelina den Braver[1], Karin Johanna Borgonjen-van den Berg[2], Femke Rutters[1], Amber van der Heijden[3], Joline Wilhelma Johanna Beulens[1,4]

[1] *Amsterdam UMC, Vrije Universiteit Amsterdam, Department of Epidemiology and Data Science, Amsterdam Public Health Research Institute, Amsterdam, The Netherlands*

[2] *Wageningen University and Research, Department of Agrotechnology and Food Sciences, Division of Human Nutrition and Health, Wageningen, The Netherlands*

[3] *Amsterdam UMC, Vrije Universiteit Amsterdam, Department of General Practice, Amsterdam Public Health Research Institute, Amsterdam, The Netherlands*

[4] *Julius Center for Health Sciences and Primary Care, University Medical Center Utrecht, Utrecht, The Netherlands*

Corresponding author: ECM Bartels (e-mail: e.c.m.bartels@amsterdamumc.nl)

Journal: EJON

Online Resource 1: Overview calculation methods, cut-off and threshold values per component of the DHD15-index

Component	Dutch dietary guidelines 2015	Minimum score (= 0 points, threshold value)	Maximum score (= 10 points, cut-off value)
Adequacy component			
Vegetables	Eat at least 200 g of vegetables daily	0 g/d	≥200 g/d
Fruits	Eat at least 200 g of fruit daily	0 g/d	≥200 g/d
Wholegrain products	Eat at least 90 g of wholegrain products daily	0 g/d	≥90 g/d
Legumes	Eat legumes weekly	0 g/d	≥10 g/d
Nuts	Eat at least 15 g of unsalted nuts daily	0 g/d	≥15 g/d
Fish	Eat one serving of fish weekly, preferably oily fish	0 g/d	≥15 g/d
Tea	Drink three cups of black or green tea daily	0 g/d	≥450 g/d
Moderation component			
Red meat	Limit consumption of red meat	≥100 g/d	≤45 g/d
Processed meat	Limit consumption of processed meat	≥50 g/d	0 g/d
Sugar-sweetened beverages and fruit juices	Limit consumption of sweetened beverages and fruit juices	≥250 g/d	0 g/d
Alcohol	If alcohol is consumed at all, intake should be limited to one Dutch unit (10 g ethanol) daily	Women: ≥20 g ethanol/d<br>Men: ≥30 g ethanol/d	Women: ≤10 g ethanol/d<br>Men: ≤10 g ethanol/d
Sodium	Limit consumption of table salt to 6 g daily	≥3.8 g Na/d	≤1.9 g Na/d
Optimum component			
Dairy	Eat a few portions of dairy products daily, including milk or yoghurt	0 g/d OR ≥750 g/d	300-450 g/d
Ratio component			
Fats/oils	Replace butter, hard margarines and cooking fats by soft margarines, liquid cooking fats and vegetable oils	No consumption of soft margarines, liquid cooking fats and vegetable oils OR Ratio of liquid cooking fats to solid cooking fats ≤ 0.6	No consumption of butter, hard margarines and cooking fats OR Ratio of liquid cooking fats to solid cooking fats ≥ 13
Wholegrain products	Replace refined cereal products by wholegrain products	No consumption of wholegrain products OR Ratio of whole grains to refined grains ≤ 0.7	No consumption of refined products OR Ratio of whole grains to refined grains ≥ 11
Quality component			
Coffee	Replace unfiltered coffee by filtered coffee	Any consumption of unfiltered coffee	Consumption of only filtered coffee OR No coffee consumption

Formulas per component for calculating DHD15 food group scores:

Adequacy score = $\frac{\text{intake}}{\text{cut−off value}} * 10$, Moderation score = $10 - \frac{\text{intake − cut−off value}}{\text{threshold value − cut−off value}} * 10$, Optimum score lower intakes = $10 - \frac{\text{intake}}{\text{cut−off value}} * 10$, Optimum score higher intakes = $10 - \frac{\text{intake − cut−off value}}{\text{threshold value−cut−off value}} * 10$, Ratio score = $\frac{\text{intake ratio − threshold value}}{\text{cut−off value− threshold value}}$
