# Supplementary material for: Adherence to the Dutch healthy diet index and change in glycemic control and cardiometabolic markers in people with type 2 diabetes
Source: Eur J Nutr. 2022 Mar 14;61(5):2761–73. doi: 10.1007/s00394-022-02847-6 (PMC9279194; doi:10.1007/s00394-022-02847-6)
Supplement: Supplementary file 2 — Supplementary file2 (PDF 497 KB) [file 394_2022_2847_MOESM2_ESM.pdf]

Adherence to the Dutch Healthy Diet index and change in glycemic control and cardiometabolic markers in people with type 2 diabetes

Ehlana Catharina Maria Bartels[1], Nicolette Roelina den Braver[1], Karin Johanna Borgonjen-van den Berg[2], Femke Rutters[1], Amber van der Heijden[3], Joline Wilhelma Johanna Beulens[1,4]

[1] Amsterdam UMC, Vrije Universiteit Amsterdam, Department of Epidemiology and Data Science, Amsterdam Public Health Research Institute, Amsterdam, The Netherlands

[2] Wageningen University and Research, Department of Agrotechnology and Food Sciences, Division of Human Nutrition and Health, Wageningen, The Netherlands

[3] Amsterdam UMC, Vrije Universiteit Amsterdam, Department of General Practice, Amsterdam Public Health Research Institute, Amsterdam, The Netherlands

[4] Julius Center for Health Sciences and Primary Care, University Medical Center Utrecht, Utrecht, The Netherlands

Corresponding author: ECM Bartels (e-mail: e.c.m.bartels@amsterdamumc.nl)

Journal: EJON

Online Resource 2: Baseline characteristics included and excluded participants

Supplementary table 1 *Baseline characteristics of the included and excluded participants presented as mean ± SD, median (IQR), or n (%)*

Participant characteristic	Included participants (n = 1202)	Excluded participants (n = 347)
Sex (male)	751 (62.5%)	222 (64.0%)
Age (years)	68.7 ± 9.0	67.5 ± 10.7
Diabetes duration (years)	12.8 ± 5.9	13.2 ± 6.6
Education		
Low	356 (30.1%)	110 (32.7%)
Middle	565 (47.8%)	157 (46.7%)
High	260 (22.0%)	69 (20.5%)
Employment status (employed)	362 (31.8%)	58 (35.4%)
Smoking		
Current	123 (10.3%)	64 (18.4%)
Former	683 (56.9%)	172 (49.6%)
Never	394 (32.8%)	111 (32.0%)
Physical activity (hours/week)	1.4 (2.3)	1.0 (2.3)
Glucose-lowering medication		
No medication	199 (16.6%)	54 (15.6%)
One OHA	356 (29.6%)	103 (29.7%)
≥ Two OHA	286 (23.8%)	80 (23.1%)
Only insulin	59 (4.9%)	24 (6.9%)
OHA + insulin	302 (25.1%)	86 (24.7%)
Total energy intake (kcal/day)	2140 ± 733	2395 ± 1367
DHD15-index score	72.0 ± 14.9	65.2 ± 14.9
HbA1c (mmol/mol)	53.8 ± 11.7	55.8 ± 15.8
Fasting glucose (mmol/L)	8.6 ± 2.1	9.0 ± 3.0
HDL cholesterol (mmol/L), women	1.4 ± 0.4	1.4 ± 0.5
HDL cholesterol (mmol/L), men	1.2 ± 0.3	1.2 ± 0.3
LDL cholesterol (mmol/L)	2.2 ± 0.9	2.2 ± 0.9
Cholesterol ratio	3.3 (1.5)	3.4 (1.5)
Systolic blood pressure (mm Hg)	140.7 ± 20.3	139.7 ± 20.2
Diastolic blood pressure (mm Hg)	77.8 ± 8.0	78.6 ± 8.4
eGFR (ml/min)	73.9 ± 18.2	75.5 ± 22.9
BMI (kg/m^2)	29.6 ± 5.2	29.5 ± 5.1

OHA: Oral hypoglycemic agents, DHD15: Dutch Healthy Diet index 2015, HbA1c: hemoglobin A1c, LDL: low-density lipoprotein, HDL: high-density lipoprotein, eGFR: estimated glomerular filtration rate, BMI: body mass index.
