# Supplementary material for: Adherence to the Dutch healthy diet index and change in glycemic control and cardiometabolic markers in people with type 2 diabetes
Source: Eur J Nutr. 2022 Mar 14;61(5):2761–73. doi: 10.1007/s00394-022-02847-6 (PMC9279194; doi:10.1007/s00394-022-02847-6)
Supplement: Supplementary file 3 — Supplementary file3 (PDF 625 KB) [file 394_2022_2847_MOESM3_ESM.pdf]

Adherence to the Dutch Healthy Diet index and change in glycemic control and cardiometabolic markers in people with type 2 diabetes

Ehlana Catharina Maria Bartels[1], Nicolette Roelina den Braver[1], Karin Johanna Borgonjen-van den Berg[2], Femke Rutters[1], Amber van der Heijden[3], Joline Wilhelma Johanna Beulens[1,4]

[1] *Amsterdam UMC, Vrije Universiteit Amsterdam, Department of Epidemiology and Data Science, Amsterdam Public Health Research Institute, Amsterdam, The Netherlands*

[2] *Wageningen University and Research, Department of Agrotechnology and Food Sciences, Division of Human Nutrition and Health, Wageningen, The Netherlands*

[3] *Amsterdam UMC, Vrije Universiteit Amsterdam, Department of General Practice, Amsterdam Public Health Research Institute, Amsterdam, The Netherlands*

[4] *Julius Center for Health Sciences and Primary Care, University Medical Center Utrecht, Utrecht, The Netherlands*

Corresponding author: ECM Bartels (e-mail: e.c.m.bartels@amsterdamumc.nl)

Journal: EJON

Online Resource 3: HbA1c (%) analyses

Supplementary table 2 *HbA1c (%) at baseline (n = 1202) presented as mean ± SD.*

Participant characteristic	Total population	DHD15-index tertiles		
		T1 (n = 400)<br>< 65.6	T2 (n = 401)<br>65.6 – 78.4	T3 (n = 401)<br>> 78.4
HbA1c (%)	7.1 ± 1.1	7.1 ± 1.0	7.0 ± 1.1	7.1 ± 1.1

DHD15: Dutch Healthy Diet index 2015, HbA1c: hemoglobin A1c.

Supplementary table 3 *Change in HbA1c (%) after two years of follow-up presented as mean ± SD (n = 916)*

Cardiometabolic parameter	Total population	DHD15-index tertiles		
		T1 (n = 400)<br>< 65.6	T2 (n = 401)<br>65.6 – 78.4	T3 (n = 401)<br>> 78.4
Δ HbA1c (%)	0.30 ± 1.02	0.26 ± 1.12	0.31 ± 0.94	0.33 ± 1.00

HbA1c: hemoglobin A1c, Δ: change in HbA1c after two years of follow-up.

Supplementary table 4 *Association between adherence to the DHD15-index at baseline and change in HbA1c (%) (n=1202) [a]*

HbA1c (%)	T1	T2		T3		*P* for	Continuous (per 10 point)	
		β	95% CI	β	95% CI	trend	β	95% CI
Model 1	Ref	-0.04	-0.18; 0.09	0.03	-0.11; 0.17	0.65	0.01	-0.03; 0.05
Model 2	Ref	-0.01	-0.15; 0.13	0.06	-0.09; 0.20	0.44	0.02	-0.02; 0.06
Model 3	Ref	0.01	-0.12; 0.15	0.10	-0.04; 0.24	0.17	0.03	-0.01; 0.07
Model 4	Ref	-0.01	-0.13; 0.11	0.05	-0.07; 0.18	0.40	0.02	-0.02; 0.05

β*:* unstandardized regression coefficient, CI: confidence interval, HbA1c: hemoglobin A1c.
[a]participant ID included as random intercept.
Model 1: Adjusted for age, sex and total energy intake.
Model 2: Additionally adjusted for education, employment status, smoking and physical activity.
Model 3: Additionally adjusted for body mass index.
Model 4: Model 2 additionally adjusted for glucose-lowering medication.

Supplementary table 5 *Baseline HbA1c (%) of the included and excluded participants presented as mean ± SD.*

Participant characteristic	Included participants (n = 1202)	Excluded participants (n = 347)
HbA1c (%)	7.1 ± 1.1	7.3 ± 1.4

HbA1c: hemoglobin A1c.

Supplementary table 6 *Complete case analyses for the association between adherence to the DHD15-index at baseline and change in HbA1c (%) [a, b]*

HbA1c (%)	T1	T2		T3		*P* for	Continuous (per 10 point)	
		β	95% CI	β	95% CI	trend	β	95% CI
Main analyses (n=1202)	Ref	-0.01	-0.15; 0.13	0.06	-0.09; 0.20	0.44	0.02	-0.02; 0.06
CCA (n=608)	Ref	0.03	-0.16; 0.21	0.03	-0.16; 0.22	0.76	0.00	-0.06; 0.05

β*:* unstandardized regression coefficient*,* CI: confidence interval, CCA: complete case analysis, HbA1c: hemoglobin A1c.
[a]participant ID included as random intercept.
[b]model 2 presented: adjusted for age, sex, total energy intake, education, employment status, smoking and physical activity.

Supplementary table 7 *Association between adherence to the DHD15-index at baseline and change in HbA1c (%), excluding under-reporters [a, b]*

HbA1c (%)	T1	T2		T3		*P* for trend	Continuous (per 10 point)	
		β	95% CI	β	95% CI		β	95% CI
Main analyses (n=1202)	Ref	-0.01	-0.15; 0.13	0.06	-0.09; 0.20	0.44	0.02	-0.02; 0.06
Excl. under-reporters (n=1079)	Ref	0.02	-0.13; 0.17	0.07	-0.08; 0.22	0.37	0.03	-0.02; 0.07

β: unstandardized regression coefficient, CI: confidence interval, HbA1c: hemoglobin A1c.
[a]participant ID included as random intercept.
[b]model 2 presented: adjusted for age, sex, total energy intake, education, employment status, smoking and physical activity.

Supplementary table 8 *Association between adherence to the DHD15-index at baseline and change in HbA1c (%), excluding alcohol (n=1202) [a, b]*

HbA1c (%)	T1	T2		T3		*P* for trend	Continuous (per 10 point)	
		β	95% CI	β	95% CI		β	95% CI
DHD15-index	Ref	-0.01	-0.15; 0.13	0.06	-0.09; 0.20	0.44	0.02	-0.02; 0.06
DHD15-index excl. alcohol	Ref	-0.06	-0.20; 0.08	-0.03	-0.17; 0.12	0.73	-0.02	-0.06; 0.03

β*:* unstandardized regression coefficient*,* CI: confidence interval, DHD15: Dutch Healthy Diet index 2015, HbA1c: hemoglobin A1c.
[a]participant ID included as random intercept.
[b]model 2 presented: adjusted for age, sex, total energy intake, education, employment status, smoking and physical activity.
