# Supplementary material for: Adherence to the Dutch healthy diet index and change in glycemic control and cardiometabolic markers in people with type 2 diabetes
Source: Eur J Nutr. 2022 Mar 14;61(5):2761–73. doi: 10.1007/s00394-022-02847-6 (PMC9279194; doi:10.1007/s00394-022-02847-6)
Supplement: Supplementary file 4 — Supplementary file4 (PDF 621 KB) [file 394_2022_2847_MOESM4_ESM.pdf]

Adherence to the Dutch Healthy Diet index and change in glycemic control and cardiometabolic markers in people with type 2 diabetes

Ehlana Catharina Maria Bartels[1], Nicolette Roelina den Braver[1], Karin Johanna Borgonjen-van den Berg[2], Femke Rutters[1], Amber van der Heijden[3], Joline Wilhelma Johanna Beulens[1,4]

[1] *Amsterdam UMC, Vrije Universiteit Amsterdam, Department of Epidemiology and Data Science, Amsterdam Public Health Research Institute, Amsterdam, The Netherlands*

[2] *Wageningen University and Research, Department of Agrotechnology and Food Sciences, Division of Human Nutrition and Health, Wageningen, The Netherlands*

[3] *Amsterdam UMC, Vrije Universiteit Amsterdam, Department of General Practice, Amsterdam Public Health Research Institute, Amsterdam, The Netherlands*

[4] *Julius Center for Health Sciences and Primary Care, University Medical Center Utrecht, Utrecht, The Netherlands*

Corresponding author: ECM Bartels (e-mail: e.c.m.bartels@amsterdamumc.nl)

Journal: EJON

Online Resource 4: Complete case analyses

Supplementary table 9 *Complete case analyses for the association between adherence to the DHD15-index at baseline and change in cardiometabolic parameters (n=608)* [a, c]

	T1		T2		T3	*P* for	Continuous (per 10 point)	
		β	95% CI	β	95% CI	trend	β	95% CI
**HbA1c (mmol/mol)**	**T1**		**T2**		**T3**	***P* for trend**	**Continuous (per 10 point)**	
		β	95% CI	β	95% CI		β	95% CI
Main analyses (n=1202)	Ref	-0.11	-1.61; 1.39	0.62	-0.94; 2.19	0.44	0.17	-0.27; 0.61
CCA (n=608)	Ref	0.27	-1.76; 2.31	0.33	-1.79; 2.46	0.76	-0.03	-0.62; 0.56
**Fasting glucose (mmol/L)**	**T1**	β	**T2** 95% CI	β	**T3** 95% CI	***P* for trend**	**Continuous (per 10 point)** β	95% CI
Main analyses (n=1202)	Ref	-0.29	-0.55; -0.03*	-0.14	-0.41; 0.13	0.30	-0.05	-0.13; 0.03
CCA (n=608)	Ref	-0.41	-0.75; -0.06*	-0.26	-0.61; 0.10	0.17	-0.09	-0.19; 0.01
**HDL cholesterol (mmol/L), women**	**T1**	β	**T2** 95% CI	β	**T3** 95% CI	***P* for trend**	**Continuous (per 10 point)** β	95% CI
Main analyses (n=1202)	Ref	0.00	-0.10; 0.09	0.08	-0.01; 0.17	0.06	0.02	0.00; 0.05
CCA (n=608)	Ref	0.07	-0.08; 0.23	0.14	-0.01; 0.29	0.06	0.04	0.00; 0.08
**HDL cholesterol (mmol/L), men**	**T1**	β	**T2** 95% CI	β	**T3** 95% CI	***P* for trend**	**Continuous (per 10 point)** β	95% CI
Main analyses (n=1202)	Ref	-0.03	-0.08; 0.02	-0.01	-0.07; 0.04	0.58	-0.01	-0.03; 0.01
CCA (n=608)	Ref	-0.02	-0.09; 0.04	-0.04	-0.11; 0.04	0.31	-0.01	-0.03; 0.01
**LDL cholesterol (mmol/L)**	**T1**	β	**T2** 95% CI	β	**T3** 95% CI	***P* for trend**	**Continuous (per 10 point)** β	95% CI
Main analyses (n=1202)	Ref	0.03	-0.08; 0.14	0.04	-0.08; 0.15	0.54	0.02	-0.01; 0.06
CCA (n=608)	Ref	0.08	-0.07; 0.23	0.11	-0.05; 0.26	0.19	0.05	0.01; 0.09*
**Cholesterol ratio**	**T1**	β	**T2** 95% CI	β	**T3** 95% CI	***P* for trend**	**Continuous (per 10 point)** β	95% CI
Main analyses (n=1202)	Ref	0.08	-0.07; 0.23	-0.06	-0.22; 0.10	0.44	-0.01	-0.05; 0.04
CCA (n=608)	Ref	0.19	-0.03; 0.40	0.09	-0.14; 0.31	0.46	0.02	-0.04; 0.08
**SBP (mm Hg)**	**T1**	β	**T2** 95% CI	β	**T3** 95% CI	***P* for trend**	**Continuous (per 10 point)** β	95% CI
Main analyses (n=1202)	Ref	-1.93	-4.27; 0.40	-1.17	-3.60; 1.25	0.34	-0.34	-1.02; 0.34
CCA (n=608)	Ref	-1.43	-4.53; 1.67	-0.22	-3.42; 2.99	0.91	0.02	-0.87; 0.92
**DBP (mm Hg)**	**T1**	β	**T2** 95% CI	β	**T3** 95% CI	***P* for trend**	**Continuous (per 10 point)** β	95% CI
Main analyses (n=1202)[b]	Ref	-0.41	-1.36; 0.55	-0.65	-1.64; 0.34	0.20	-0.17	-0.44; 0.11
CCA (n=608)[b]	Ref	0.30	-0.99; 1.60	-0.18	-1.50; 1.15	0.77	0.00	-0.37; 0.37
**eGFR (ml/min)**	**T1**	β	**T2** 95% CI	β	**T3** 95% CI	***P* for trend**	**Continuous (per 10 point)** β	95% CI
Main analyses (n=1202)	Ref	0.11	-2.30; 2.52	1.74	-0.76; 4.25	0.18	0.40	-0.30; 1.11
CCA (n=608)	Ref	0.64	-2.73; 4.02	3.47	-0.04; 6.98	0.05	0.76	-0.22; 1.74
**BMI (kg/m^2)**	**T1**	β	**T2** 95% CI	β	**T3** 95% CI	***P* for trend**	**Continuous (per 10 point)** β	95% CI
Main analyses (n=1202)	Ref	-0.69	-1.37; -0.01*	-1.37	-2.07; -0.66**	<0.001**	-0.41	-0.60; -0.21**
CCA (n=608)	Ref	-0.41	-1.28; 0.46	-1.08	-1.98; -0.17*	0.02*	-0.41	-0.67; -0.16**

β: unstandardized regression coefficient, CI: confidence interval, CCA: complete case analysis, HbA1c: hemoglobin A1c, LDL: low-density lipoprotein, HDL: high-density lipoprotein, SBP: systolic blood pressure, DBP: diastolic blood pressure, eGFR: estimated glomerular filtration rate, BMI: body mass index.
*p value < 0.05.
**p value < Bonferroni corrected alpha (= 0.0045).
[a]participant ID included as random intercept.
[b]sex included as random slope.
[c]model 2 presented: adjusted for age, sex, total energy intake, education, employment status, smoking and physical activity.
