# Supplementary material for: Adherence to the Dutch healthy diet index and change in glycemic control and cardiometabolic markers in people with type 2 diabetes
Source: Eur J Nutr. 2022 Mar 14;61(5):2761–73. doi: 10.1007/s00394-022-02847-6 (PMC9279194; doi:10.1007/s00394-022-02847-6)
Supplement: Supplementary file 5 — Supplementary file5 (PDF 621 KB) [file 394_2022_2847_MOESM5_ESM.pdf]

Adherence to the Dutch Healthy Diet index and change in glycemic control and cardiometabolic markers in people with type 2 diabetes

Ehlana Catharina Maria Bartels[1], Nicolette Roelina den Braver[1], Karin Johanna Borgonjen-van den Berg[2], Femke Rutters[1], Amber van der Heijden[3], Joline Wilhelma Johanna Beulens[1,4]

[1] Amsterdam UMC, Vrije Universiteit Amsterdam, Department of Epidemiology and Data Science, Amsterdam Public Health Research Institute, Amsterdam, The Netherlands

[2] Wageningen University and Research, Department of Agrotechnology and Food Sciences, Division of Human Nutrition and Health, Wageningen, The Netherlands

[3] Amsterdam UMC, Vrije Universiteit Amsterdam, Department of General Practice, Amsterdam Public Health Research Institute, Amsterdam, The Netherlands

[4] Julius Center for Health Sciences and Primary Care, University Medical Center Utrecht, Utrecht, The Netherlands

Corresponding author: ECM Bartels (e-mail: e.c.m.bartels@amsterdamumc.nl)

Journal: EJON

Online Resource 5: Analyses excluding under-reporters

Supplementary table 10 *Association between adherence to the DHD15-index at baseline and change in cardiometabolic parameters, excluding under-reporters (n=1079)* [a, c]

**HbA1c (mmol/mol)**	**T1**	**T2**		**T3**		***P* for trend**	**Continuous (per 10 point)**	
		β	95% CI	β	95% CI		β	95% CI
Main analyses (n=1202)	Ref	-0.11	-1.61; 1.39	0.62	-0.94; 2.19	0.44	0.17	-0.27; 0.61
Excl. under-reporters (n=1079)	Ref	0.20	-1.40; 1.80	0.75	-0.90; 2.41	0.37	0.27	-0.19; 0.73
**Fasting glucose (mmol/L)**	**T1**	**T2**		**T3**		***P* for trend**	**Continuous (per 10 point)**	
		β	95% CI	β	95% CI		β	95% CI
Main analyses (n=1202)	Ref	-0.29	-0.55; -0.03*	-0.14	-0.41; 0.13	0.30	-0.05	-0.13; 0.03
Excl. under-reporters (n=1079)	Ref	-0.28	-0.55; 0.00*	-0.17	-0.45; 0.11	0.25	-0.05	-0.13; 0.03
**HDL cholesterol (mmol/L), women**	**T1**	**T2**		**T3**		***P* for trend**	**Continuous (per 10 point)**	
		β	95% CI	β	95% CI		β	95% CI
Main analyses (n=1202)	Ref	0.00	-0.10; 0.09	0.08	-0.01; 0.17	0.06	0.02	0.00; 0.05
Excl. under-reporters (n=1079)	Ref	0.00	-0.10; 0.11	0.07	-0.03; 0.17	0.11	0.02	-0.01; 0.05
**HDL cholesterol (mmol/L), men**	**T1**	**T2**		**T3**		***P* for trend**	**Continuous (per 10 point)**	
		β	95% CI	β	95% CI		β	95% CI
Main analyses (n=1202)	Ref	-0.03	-0.08; 0.02	-0.01	-0.07; 0.04	0.58	-0.01	-0.03; 0.01
Excl. under-reporters (n=1079)	Ref	-0.03	-0.09; 0.02	-0.01	-0.08; 0.05	0.56	-0.01	-0.03; 0.01
**LDL cholesterol (mmol/L)**	**T1**	**T2**		**T3**		***P* for trend**	**Continuous (per 10 point)**	
		β	95% CI	β	95% CI		β	95% CI
Main analyses (n=1202)	Ref	0.03	-0.08; 0.14	0.04	-0.08; 0.15	0.54	0.02	-0.01; 0.06
Excl. under-reporters (n=1079)	Ref	0.05	-0.07; 0.17	0.03	-0.09; 0.16	0.60	0.02	-0.01; 0.06
**Cholesterol ratio**	**T1**	**T2**		**T3**		***P* for trend**	**Continuous (per 10 point)**	
		β	95% CI	β	95% CI		β	95% CI
Main analyses (n=1202)	Ref	0.08	-0.07; 0.23	-0.06	-0.22; 0.10	0.44	-0.01	-0.05; 0.04
Excl. under-reporters (n=1079)	Ref	0.08	-0.08; 0.24	-0.07	-0.24; 0.09	0.38	-0.01	-0.05; 0.04
**SBP (mm Hg)**	**T1**	**T2**		**T3**		***P* for trend**	**Continuous (per 10 point)**	
		β	95% CI	β	95% CI		β	95% CI
Main analyses (n=1202)	Ref	-1.93	-4.27; 0.40	-1.17	-3.60; 1.25	0.34	-0.34	-1.02; 0.34
Excl. under-reporters (n=1079)	Ref	-1.99	-4.45; 0.47	-0.77	-3.32; 1.78	0.56	-0.18	-0.89; 0.53
**DBP (mm Hg)**	**T1**	**T2**		**T3**		***P* for trend**	**Continuous (per 10 point)**	
		β	95% CI	β	95% CI		β	95% CI
Main analyses (n=1202)[b]	Ref	-0.41	-1.36; 0.55	-0.65	-1.64; 0.34	0.20	-0.17	-0.44; 0.11
Excl. under-reporters (n=1079)[b]	Ref	-0.70	-1.70; 0.30	-0.61	-1.64; 0.42	0.26	-0.14	-0.43; 0.14
**eGFR (ml/min)**	**T1**	**T2**		**T3**		***P* for trend**	**Continuous (per 10 point)**	
		β	95% CI	β	95% CI		β	95% CI
Main analyses (n=1202)	Ref	0.11	-2.30; 2.52	1.74	-0.76; 4.25	0.18	0.40	-0.30; 1.11
Excl. under-reporters (n=1079)	Ref	0.07	-2.56; 2.70	2.17	-0.56; 4.90	0.12	0.51	-0.25; 1.27
**BMI (kg/m^2)**	**T1**	**T2**		**T3**		***P* for trend**	**Continuous (per 10 point)**	
		β	95% CI	β	95% CI		β	95% CI
Main analyses (n=1202)	Ref	-0.69	-1.37; -0.01*	-1.37	-2.07; -0.66**	<0.001**	-0.41	-0.60; -0.21**
Excl. under-reporters (n=1079)	Ref	-0.54	-1.23; 0.16	-1.33	-2.05; -0.60**	<0.001**	-0.38	-0.58; -0.18**

β*:* unstandardized regression coefficient, CI: confidence interval, HbA1c: hemoglobin A1c, LDL: low-density lipoprotein, HDL: high-density lipoprotein, SBP: systolic blood pressure, DBP: diastolic blood pressure, eGFR: estimated glomerular filtration rate, BMI: body mass index.

**p* value < 0.05.

***p* value < Bonferroni corrected alpha (= 0.0045).

[a]participant ID included as random intercept.

[b]sex included as random slope.

[c]model 2 presented: adjusted for age, sex, total energy intake, education, employment status, smoking and physical activity.
